# Teacher Evaluation of an Experiential Vegetable Education Program for Australian Primary Schools: Does Face-to-Face Training Add Value above Digital Training?

**DOI:** 10.3390/nu13051648

**Published:** 2021-05-13

**Authors:** Astrid A. M. Poelman, Maeva Cochet-Broch, Janne Beelen, Bonnie Wiggins, Jessica E. Heffernan, David N. Cox

**Affiliations:** 1 Sensory and Consumer Science, CSIRO Agriculture and Food, North Ryde, NSW 2113, Australia; maeva.broch@csiro.au (M.C.-B.); janne.beelen@csiro.au (J.B.); jess.heffernan@csiro.au (J.E.H.); 2 Public Health Nutrition, CSIRO Health and Biosecurity, Adelaide, SA 5000, Australia; bonnie.wiggins@csiro.au (B.W.); david.cox@csiro.au (D.N.C.)

**Keywords:** vegetable, primary school, implementation science, child health, acceptance, schoolteacher, process evaluation, cluster RCT

## Abstract

The teacher-led implementation of healthy eating programs in schools is cost-effective and potentially impactful. Teacher acceptability is important for uptake; however, process evaluations are scarce. This study evaluated the effect of two intensities of teacher training on the evaluation of a vegetable education program for Australian primary schools by teachers. The teachers (*n* = 65) who implemented the program as part of a cluster RCT (25 schools in two states, New South Wales and South Australia) received either low- (provision with materials and online training) or high (additional face-to-face (F2F) training)-intensity training prior to implementing a 5-week vegetable education program. They evaluated the acceptability of a digital training module and program by indicating the level of agreement with 15 and 18 statements, respectively, using 5-point Likert scales. The average item scores ranged from 3.0 to 4.2. All but one item, including student engagement, alignment to the curriculum and intent for reuse of the program, had a rounded average or median score of 4. The level of training intensity did not impact the teacher acceptability ratings. In conclusion, the teacher acceptability was good, and additional F2F training does not add value above the solely digital training of the teachers.

## 1. Introduction

Schools provide an important and equitable opportunity to support healthy eating amongst students [1,2]. From a public health perspective, the rationale for school-based programs to support healthy eating is evident; schools reach all students, regardless of background, thereby provide opportunities to improve children’s population health and bridge health inequality gaps [3]. In addition, they provide opportunities for the development of skills, knowledge and attitudes towards healthy eating behaviours [1], irrespective of the family dynamics [4] and other parental barriers [5,6]. A strong evidence base in behavioural outcomes is important for government health promotion agencies and policy-makers to justify the endorsement and implementation of programs on a large scale [7].

The evaluations of nutrition and other health-promoting programs most often focus on impact and/or effect evaluations, whereas process evaluations are less frequently undertaken [8,9,10]. Process evaluations offer insights into whether the program is implemented as intended and whether the program is perceived as acceptable and appropriate by participants [11,12]. Process evaluations allow to make modifications prior to undertaking large-scale effect studies and/or the commencement of full implementation and, in this way, maximizes the potential success of a program [11].

Acceptability (appreciation) is perceived to be amongst the most important indicators of process evaluations [12]. To maximize the uptake and adoption, it is critically important to ensure the teacher acceptability of healthy eating classroom resources. School curricula are crowded, and teachers are stressed and lack time [13,14]. Moreover, teachers have considerable influence on deciding which materials and programs are being used in order to meet the curriculum standards [15].

Effect and teacher acceptability evaluations were undertaken on a newly developed vegetable education resource for primary schools to increase children’s vegetable acceptance and willingness to try [16,17]. Children’s intake of vegetables is far below the recommended intake in Australia, as in most other Western countries [18], with a low (sensory) acceptance of vegetables a critical barrier [19,20]. The vegetable education program Taste & Learn™ consists of a teacher-led classroom-based program for Australian primary schools to increase children’s enjoyment of vegetables. The scientific framework is based on evidence from food and vegetable preference development [19,20] and sensory education [21,22]; the key elements are building exposure and familiarity with vegetables through tasting, the verbalization of sensations, science experiments and a positive and fun environment. The program consists of 5 × 1-h lessons for the three different stages of primary school. Vegetables are tasted in each lesson, and the program is aligned to the Australian primary school curriculum [23]. The program was initially evaluated in a pre-post-pilot study in four NSW schools. The results demonstrated that the program positively influenced the mediating factors associated with vegetable consumption amongst the students, including vegetable knowledge and acceptance [17]. A quantitative teacher acceptability survey showed that the teachers positively evaluated most aspects of the program, including student engagement and alignment to the curriculum [16]. However, preparation effort for preparing fresh vegetables was seen as considerable [16]. The interviews with the teachers further showed that the lesson program was very content-dense (unpublished data).

Information from the pilot study on the effect and evaluation of teacher acceptability was used to refine the vegetable education program and its supporting materials. Considerable attention was given to minimise the preparation efforts and specifying the produce quantities needed for each lesson to manage the teacher’s expectations; this was done by calculating the minimal required quantities needed for tastings and translate those to the amount of vegetables needed on a classroom level (e.g., one small broccoli floret per student for tasting, requiring one medium head of broccoli per classroom) and reducing the variety of foods offered in lessons where a vegetable meal was prepared. Other changes included a reduction in the content density of the lessons, whilst simultaneously ensuring that the content retained both a behavioural change focus, as well as strong curriculum alignment. The 5E pedagogical framework [24] was changed to move through the five steps of Engage, Explore, Explain, Elaborate and Evaluate throughout the five lessons rather than in each individual lesson. Additionally, an online training module for teachers was developed.

A cluster randomised controlled trial (cluster RCT) amongst 25 schools involving 1639 students was subsequently undertaken to measure the effects on the behavioural outcomes [25]. This study used two intervention arms that differed in the level of intensities of the training of the teacher, low (provision with materials and online training) and high (additional face-to-face (F2F) training), therefore differing in cost structures (one-off vs. ongoing costs) and impacting the potential scalability of the intervention. The results showed that the program increased the students’ knowledge, verbalization ability, vegetable acceptance, behavioural intentions, willingness to taste and consumption of new vegetables during the post-test, with their knowledge sustained at the 3-month follow-up. No difference was found between the level of intensity of training on the student outcomes [25].

The current study was a process evaluation undertaken as part of the cluster RCT, which focused on the teacher acceptability of the program. The aims were two-fold: (1) to compare the effect of a low- and high-intensity training program on the teacher acceptability of a vegetable education resource and (2) to compare the teacher acceptability results from the modified version of the vegetable education resource with the previous version of the resource, to determine whether the content changes affected the teacher acceptability. The results will be used to support implementation on a larger scale and identify if there are areas for further improvement.

## 2. Materials and Methods

### 2.1. Participants

Eligible participants in this study were primary school teachers who implemented the vegetable education program Taste & Learn™ in their classroom as part of a cluster randomised controlled trial to measure behavioural outcomes on students [25]. The cluster-randomised controlled trial was undertaken in 25 Australian schools, comprising 19 intervention schools where teachers received a high- (*n* = 10) or a low (*n* = 9)-intensity version of teaching training prior to implementation of the program. Additionally, 6 control schools received no training and continued to implement their regular school curriculum. Teachers in both intervention arms were eligible to take part in the teacher evaluation reported here.

Research was undertaken in two state capital cities: Sydney, New South Wales (NSW) and Adelaide, South Australia (SA) to determine whether any differences existed between states in teacher appreciation of the intervention due to variations in curriculum implementation and health policy by states. Until recently, each state in Australia had its own school curriculum, with state differences in the curricula having historical, geographical or demographic roots [26]. Australia moved to a national school curriculum in 2014, but implementation is at the state level. Each state has their own Department of Education, and slight differences in implementation and priorities exist. For example, NSW uses a NSW curriculum that is based on the Australian curriculum [27], whereas SA uses the Australian curriculum. In addition, differences between states exist in nutrition programs run in schools.

Ethical approval for this study was provided by the CSIRO Human Research Ethics Committee (HREC24/2016), the NSW Department of Education and Communities (SERAP2017036) and the SA Department for Education (2018-0032). This trial was registered with the Australian New Zealand Clinical Trials Registry (ACTRN12620000392965).

### 2.2. Teacher Training and Vegetable Education Program

The vegetable education program Taste & Learn™ was described in the Introduction. Detailed lesson plans were provided to teachers, which included suggested vegetables for each lesson. Schools were responsible for sourcing the vegetables themselves, and they were reimbursed upon the production of receipts. Further details of this program have been reported elsewhere [25].

Prior to implementing the program in their classrooms, the teachers received one of two forms of training: (1) Low-intensity training: teacher received written lesson materials and an implementation manual, as well as an individual link to a Learning Management System (LMS) to undertake an online training module, which took around 20 min to complete. Adherence was monitored through the LMS platform. The implementation manual and online training module both covered the objectives of the program, theoretical information on the senses and the development of food acceptance in children and practical information to implement the program. The implementation manual also contained detailed information on alignment to the Australian curriculum. (2) High-intensity training: teachers received lesson materials, manual and online training modules, as in the low-intensity training, but additional interactive face-to-face (F2F) training was provided. F2F training (45 min) was delivered by research staff involved in the study and contained information on the same elements as delivered through the online training and written resources. In addition, implementation plans for their school were discussed with the staff. Adherence to the intervention was monitored through phone contact with the ‘champion’ for the study in each intervention school (both low and high) and through the reimbursement of costs for materials to implement the program.

### 2.3. Outcome Measures

Participants were sent a link by email to take part in an online survey (SurveyGizmo) shortly after they implemented the vegetable education program in their classroom. NSW teachers implemented the program in school term 2 (April–June 2018) and were sent the survey link at the end of June 2018. SA teachers implemented the program in school term 3 (July–mid-September 2018) and were sent the survey link mid-September 2018. This was at the same time that the post-test student behavioural data [25] were collected. The survey evaluated both the online training module that teachers were provided access to and the vegetable education program they implemented by rating their level of agreement with the statements using five-point Likert scales (1 = strongly disagree to 5 = strongly agree). The evaluation of the online training module consisted of 15 statements (Table 1), 14 of which were based on the Learning Object Review Instrument (LORI), a framework for evaluating the quality of multimedia learning resources [28]. This framework consists of 9 key dimensions, of which 6 were relevant to the online training module and for which statements were developed a priori: content quality, learning goal alignment, motivation, presentation design, interaction usability and reusability. The other dimensions of the LORI framework were deemed as not applicable (feedback and adaptation) or not relevant (accessibility and standard compliance) to the online module. A further statement on the duration of the module was included. Participants could also provide comments.

The vegetable education program was evaluated using 18 statements (Table 2), covering 8 out of 9 key dimensions of the LORI framework [28]. In addition, feasibility was added as an additional construct, as it was deemed important for teacher uptake. The statements covered various aspects of the suitability and relevance for students and the suitability of materials and alignment to the curriculum, as well as whether the teacher would reuse the program and recommend it to other teachers. Eleven of the 18 statements were the same as that used in the teacher evaluation of a previous version of the program [16], so that results could be directly compared. In addition, participants provided an overall score (out of 10) for the program. As open questions, teachers were asked what the best features of the program were and what features could be improved.

### 2.4. Data Analysis

Data analysis was conducted using SPSS (IBM Corp. Released 2017. IBM SPSS Statistics for Windows, Version 25.0. Armonk, NY: IBM Corp.). A value of *p* < 0.05 was used as a measure for statistical significance.

For the online training module and vegetable education program separately, first, internal consistency of the items pertaining to the same construct (e.g., learning goals and content quality) were calculated using Cronbach’s alpha. An average score was calculated for constructs with sufficient internal consistency (Cronbach’s alpha > 0.70). Where the internal consistency was lower, the individual items were retained and median values reported because of skewed distributions. To determine if there were differences in the responses between teachers from different intervention arms and states, the univariate analysis of variance (ANOVA) was conducted with the dimension ratings as dependent variables and with the training intensity (low/high) and state (NSW/SA) as the independent factors. Nonparametric (Mann–Whitney *U*) tests were applied to the single-item ratings, as these variables had a skewed distribution (based on visual interpretation of the Q-Q plots).

In addition, a Mann–Whitney *U* test was conducted to compare the teachers’ acceptability ratings from the pilot program [16] to the current program. This analysis was undertaken to determine whether changes to the content of the program and materials affected the acceptability; therefore, it was undertaken with teachers from NSW only to match the participant group of the pilot study as closely as possible [16].

## 3. Results

### 3.1. Participants

A total of 65 teachers (state: 58% NSW, 42% SA; training intensity: 57% high, 43% low) completed the survey, which was a response rate of 78% of the eligible teachers. A total of 73% of teachers in the high-intensity training arm completed the survey and 88% of teachers in the low-intensity training arm. Feedback from teachers from 17 out of 19 intervention schools was received, with an average of 3.8 ± 2.5 teachers per school. The teachers covered all the year levels in primary school, with 23% of teachers who had taught lower primary school classes (5–8 year olds), 40% middle primary school classes (8–10 year olds) and 15% upper primary school classes (10–12 year olds), and 22% taught classes from multiple stages of primary school.

### 3.2. Acceptability of Online Training Module

Seventy-eight percent of teachers (*n* = 51; state: 51% NSW, 49% SA; training intensity: 59% high, 41% low) indicated having conducted the online training module. A total of 75% of teachers in the low-intensity training arm conducted the online training module and 82% of teachers in the high-intensity training arm. There was good internal consistency for the dimensions of content quality, learning goals, motivation, presentation design and reusability (Table 3); for these dimensions, the average ratings were calculated. The dimension interaction usability had a Cronbach’s alpha of 0.69, and its items were analysed separately.

The average dimension and item scores ranged from 3.9 to 4.1. All dimensions and items had an average score (rounded to the closest whole number) or median score of 4. There were no statistically significant differences in ratings as a factor of the intervention arm (training intensity) or state (NSW/SA) (Table 3).

Open comments provided positive feedback (e.g., interesting, easy to use and informative); comments related to accessing the materials (time-consuming to download and some technical difficulties, primarily from NSW teachers where the program was rolled out first) and comments related to the content. On the latter, two teachers wished that the module provided detailed training on a lesson-by-lesson basis, whereas another teacher commented that the module was not needed, as sufficient background information was given in the lessons themselves.

### 3.3. Acceptability of Vegetable Education Program

The item scores for the vegetable education program ranged from 3.0 to 4.2 (Table 4). There was a good internal consistency for the dimensions of content quality, learning goals, motivation, accessibility and reusability (Table 4); for these dimensions, the average ratings were calculated. The dimension feasibility had a Cronbach’s alpha of 0.68, and its items were analysed separately. All but one dimension or item had an average (rounded to the closest whole number) or median rating of 4; this included ratings related to the statements of student engagement, suitability for students of all backgrounds and abilities, alignment to the curriculum, perception of the long-lasting impact on students, use of suggested vegetables and intent to reuse the program and recommend it to other teachers. One item had a median score of 3 (neutral level of the scale); this was related to the amount of preparation prior to the lesson. The overall program rating was 7.3 ± 1.9.

The statistical analysis showed that the level of intensity of training (online or additional F2F) did not affect any of the acceptability ratings of the vegetable education program by the teachers (Table 4). Differences between teachers from different states were found in four instances, i.e., in the ratings for motivation, accessibility, a good mix of materials and the duration of lessons (Table 4). The teachers from NSW rated the vegetable education program higher than teachers from SA in the constructs Motivation and Accessibility, and they were also more agreeable towards the statement that the durations of the lessons were appropriate (Table 5). The median ratings for the mix of materials were the same; however the interquartile range showed that the NSW teachers were more uniform in their ratings than the SA teachers. The teachers provided comments on the best features of the program and potential for improvement. The most commonly mentioned best features included the vegetable tastings and students trying new foods/vegetables, the high student engagement through the hands-on learning aspect and the good resources of the program overall. Further positive comments were also made about specific program aspects, particularly the last lesson (where students eat and prepare a dish together), which was very well-received, the concept of a food adventurer and the information that the program provided about vegetables. Several teachers also mentioned that the program was important in challenging preconceived ideas and allowed the students to take some risks, which they liked.

Suggestions for improvements related to the time/duration of the lessons in relation to content density, with some teachers suggesting breaking up the material into smaller lessons or reducing the amount of material. Preparation time involved for the practical aspects was also mentioned and the involvement of others suggested (e.g., teacher aid, parents and students). Some teachers also suggested adding a recording element for the students (journal/workbook/scrapbook). There were two teachers who commented that students in their first year of schooling found it difficult to come up with describing words and suggested buddy classes with older students.

### 3.4. Comparison with Pilot Evaluation

**Figure 1:**
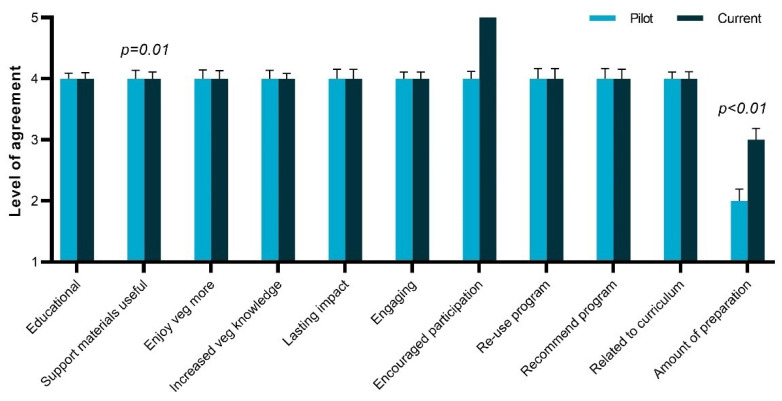
Comparison between the acceptability ratings of the previous (pilot, [16]) and current (modified) versions of the vegetable education program on 11 statements (median and SE) on a scale of 1–5 based on the responses from the New South Wales teachers (*n* = 27 in the pilot study [16] and *n* = 38 in the current study). *p-*values indicate statistically significant differences between groups.

The teacher acceptability data for 11 of 18 statements (Table 1) were also collected quantitatively in an evaluation of a previous version of the program by NSW teachers [16]. Compared to this earlier version of the program, the teachers rated the current program higher on the usefulness of the supporting materials (U = 2.5, *p* = 0.01) and the preparation efforts needed for the program (U = 2.8, *p* < 0.01) (Figure 1), with no statistical differences between the two versions for the other statements. The median value for the usefulness of the supporting materials was the same, but the interquartile range for the teachers in the current study (IQR 4-5) was higher than in the pilot study (IQR 3-4), whereas the evaluation of the preparation effort needed increased from a median value of 2 to 3 (Figure 1).

## 4. Discussion

The current study aimed to compare the effects of a low- and a high-intensity training program on the teacher acceptability of a vegetable education resource, Taste & Learn™, and compared it with the acceptability evaluations of a previous version of the program. The results showed that the vegetable education resource had good acceptability amongst the teachers, regardless of the type of training, but SA teachers were less positive about a small number of aspects of the program. Compared to a previous version of the program, the teachers evaluated the preparation efforts and materials more positively.

Acceptability of the Taste & Learn™ program by the teachers was good. Not many process evaluations of the comparable programs have been undertaken, but the acceptability of the Taste & Learn™ program was similar to the teacher acceptability of a Dutch sensory education program Taste Lessons [29]. The most appreciated elements of the Dutch Taste Lessons program by the students themselves were the taste tests and conducting experiments [29]. The current study measured the teachers’ perceptions of student engagement and found similar results, which was supported by the open comments teachers provided. In addition to being enjoyable, experiential learning activities are also amongst the most effective activities in healthy eating programs [30,31].

The current study showed that the revision of the resource materials positively contributed to the teacher acceptability of the program. Notably, the response to the statement “The amount of preparation for this program is reasonable” changed from a score of 2 (“Slightly disagree”) [16] to 3 (“Neutral”). Barriers to the implementation of a fruit and vegetable (FV) distribution program in schools include a lack of time to cut FVs [8], which may lead to serving FVs that require no or little cutting [32]. Thus, specific attention to this aspect of the program has lowered a potential barrier for uptake. Moreover, teachers mostly used the suggested vegetables for each lesson, thereby ensuring students were exposed to a broad variety of vegetables. The vegetable tastings are a critical success element to the experiential learning component of this program in terms of building vegetable enjoyment, as well as student and teacher appreciation of the program, and short of providing pre-cut vegetables, it is unlikely that further improvements can be made. The resource materials were also more positively evaluated, which shows that modifications based on the previous evaluations [16,17] were successful. It is also important to note that the teacher acceptability for all other aspects remained the same. In particular, despite the reduced contents of the resource, the perception of alignment to the curriculum remained the same.

Although there were no differences between states for the majority of the aspects, the teachers in NSW rated the program higher than SA on several aspects. The potential reasons for these differences are unclear. At the time of the study, the NSW government had an active framework of promoting healthy eating programs in schools (*Live Life Well @School*) [3] whereas SA did not, which perhaps raised the perceived importance of such programs amongst the NSW teachers; however, this remains speculative. It might also be that there are differences between the states in how teachers access training for educational programs. It is clear, however, that any differences in teacher acceptability did not impact the students’ outcomes, as no differences in their behavioural outcomes were found as a function of the states [25].

An effect evaluation of the vegetable education program showed that the level of intensity of teacher training did not affect the student outcomes [25]. The current study showed that teacher acceptability of the digital training module and the vegetable education program were also independent of the intensity of training. These results seem to favour the implementation of the program using a low(er) intensity training, as the program can be made available with no ongoing costs, e.g., through a website and implemented regardless of the geographic location. Although the high costs of F2F training are not warranted, based on the results of this study, the provision of some form of personal interaction may still be beneficial when/where possible to raise awareness of the program and support discussions around its implementation. This could take the form of a combined information sessions and training webinars, thereby lowering the costs compared to F2F and enabling a wide reach.

Two statements in the current study measured the teacher’s perceptions of specific impacts of the program on the students, i.e., positively influencing student’s vegetable knowledge and student’s vegetable acceptance. The teachers had high agreement to both statements. The intervention had positive behavioural outcome effects on the students, including knowledge and vegetable acceptance [25] consistent with the teachers’ perceptions.

The response rate of the eligible teachers was 78%. The eligibility criterion for taking part in the survey was that the teachers implemented the vegetable education program in their classroom; therefore, any teachers or schools that dropped out before implementing the vegetable education program were not eligible to take part in this survey.

There were 23 out of 106 classes (22%) in the intervention schools that dropped out after the baseline student data were collected [25]. Fifteen of those 23 classes were from the same school that initially intended to take part with the whole school but then continued with only a smaller number of classes because of time constraints [25]. Therefore, the results of this survey do not reflect the opinion of a small proportion of teachers who had access to or implemented the vegetable education program, which limits the generalizability of the findings to some extent.

## 5. Conclusions

This study demonstrated good teacher acceptability of the vegetable education program and its supporting resources. It also highlighted some further development opportunities. The online module was well-received in all aspects, but there were some technical difficulties with accessing the materials. It would also be recommended to undertake a further process evaluation of the program when the full implementation begins to determine its reach and impact on a larger scale—for example, using the RE-AIM framework [33].

## Figures and Tables

**Table 1:** Statements ^1^ used in the evaluation of the online training module and their classification according to the dimensions from the LORI framework [28].

Dimension (Cronbach’s Alpha)	Statement
Content quality (0.89)	1. The content of the online training module was relevant to teaching the vegetable education program
	2. The level of detail in the module was appropriate
Learning goals (0.89)	3. The module enhanced my knowledge about how to teach enjoyment of vegetables
	4. The module enhanced my knowledge to teach students about the senses and how to verbalise their sensations when eating vegetables
	5. The module helped me with the practical implementation of the lessons
Motivation (0.84)	6. The information provided prepared me well to teach the program to my students
	7. I found the module interesting
	8. The module motivated me to teach the program to my students
Interaction usability (0.69)	9. The training module was easy to navigate
	10. It was easy to download the resources (lesson plans, shopping lists) from the module
Presentation design (0.82)	11. The training module was appealing (visually and auditory)
	12. The presentation design (graphics, text, voice-over etc.) supported the content well
Re-usability (0.83)	13. The online training module is suitable for teachers at different levels
	14. The online training module is suitable for teachers working in different school environments
Other	15. The duration of the module was appropriate

^1^ Rated 1 to 5: 1 = strongly disagree to 5 = strongly agree.

**Table 2:** Statements ^1^ used in the evaluation of the vegetable education program and their classification according to the dimensions from the LORI framework ^2^ [28].

Dimension (Cronbach’s Alpha)	Statement ^3^
Content quality	**1. The program was educational for students**
(0.70)	**2. The program support materials were useful**
Learning goals (0.80)	**3. The program is likely to encourage students to enjoy vegetables more**
	**4. The program helped students gain knowledge of vegetables**
	**5. The program is likely to have a lasting positive impact on the students**
Motivation	**6. The program was engaging for students**
(0.84)	**7. The program encouraged student participation**
Feedback and adaptation	8. The program contained activities that allowed to gauge how much students had learned
Accessibility	9. The program was suitable for students from various backgrounds
(0.85)	10. The program was suitable for students of all abilities
Presentation design	11. There was a good mix of pictorial, text and audio materials in the teaching package
Re-usability	**12. I would use this program again**
(0.98)	**13. I would recommend this program to other teachers**
Standards	**14. The program related well to the curriculum**
Feasibility	**15. The amount of preparation for each lesson was reasonable**
(0.68)	16. The number of lessons was appropriate
	17. The duration of the lessons was appropriate
Other	18. I used the vegetables that were suggested for the lessons

^1^ Rated 1 to 5: 1 = strongly disagree to 5 = strongly agree. ^2^ Feasibility was not an original construct of the LORI framework. ^3^ Items in bold were also used in the pilot evaluation [16].

**Table 3:** Average (standard deviation (SD)) (for constructs) and median (interquartile range (IQR) (for single items) levels of agreement for various dimensions (Cronbach’s alpha) and statements by the teachers (*n* = 51) evaluating the online training module and statistical significance as a factor of intervention (high- vs. low-intensity training) and state (New South Wales vs. South Australia). The ratings ranged from 1–5.

Dimension (Cronbach’s Alpha)/Statement	Average/Median	SD/IQR	Intervention		State	
			F Value/U Value	*p*-Value	F Value/U Value	*p*-Value
Constructs						
Content quality (0.89)	4.11	0.55	0.40	0.40	2.74	0.11
Learning goals (0.89)	3.88	0.69	0.42	0.41	1.38	0.25
Motivation (0.84)	3.86	0.71	0.20	0.89	0.67	0.42
Re-usability (0.83)	3.95	0.49	0.72	0.40	3.65	0.06
Presentation design (0.82)	3.95	0.49	0.01	0.99	0.52	0.47
Single items						
The training module was easy to navigate	4	(4;4)	0.37	0.54	0.61	0.54
It was easy to download the resources from the module	4	(3;4)	0.47	0.50	1.65	0.10
The duration of the module was appropriate	4	(4;4)	0.24	0.63	1.49	0.14

**Table 4:** Average (standard deviation (SD)) (for constructs) and median (interquartile range (IQR)) (for single items) levels of agreement for various dimensions (Cronbach’s alpha) and statements by the teachers (*n* = 65) who implemented the program (across both high- and low-intensity training) evaluating the vegetable education program and statistical significance as a factor of intervention (high- vs. low-intensity training) and state (New South Wales vs. South Austalia). The ratings ranged from 1–5.

Dimension (Cronbach’s Alpha)/Statement	Average/Median	SD/IQR	Training Intensity		State	
			F Value/U Value	*p*-Value	F Value/U Value	*p*-Value
Constructs						
Content quality (0.70)	4.13	0.64	0.00	1.00	2.98	0.09
Learning goals (0.80)	4.01	0.58	0.50	0.48	0.7	0.79
Motivation (0.84)	4.21	0.64	1.49	0.23	8.62	0.005
Accessibility (0.85)	4.11	0.58	0.02	0.90	7.73	0.007
Re-usability (0.98)	3.79	0.92	2.40	0.13	0.69	0.41
Single items						
The program related well to the curriculum	4	(4;4)	0.46	0.65	0.11	0.92
The program contained activities that allowed to gauge how much students had learned	4	(3;4)	0.18	0.86	1.36	0.17
There was a good mix of pictorial, text and audio materials in the teaching package	4	(4;4)	0.90	0.37	2.25	0.02
The amount of preparation for each lesson was reasonable	3	(2;4)	1.00	0.32	0.16	0.87
The number of lessons was appropriate	4	(4;4)	1.56	0.12	1.68	0.09
The duration of the lessons was appropriate	4	(2;4)	1.42	0.16	2.61	0.009
I used the vegetables that were suggested for the lessons	4	(4;4)	0.24	0.81	0.67	0.5

**Table 5:** Average (standard deviation (SD)) for the constructs and median (interquartile range (IQR)) (for single items) level of agreement by the teachers for the dimensions (Cronbach’s alpha) and statements evaluating the vegetable education program, for which a statistical significance difference between the states was obtained.

Dimension (Cronbach’s Alpha)/Statement	New South Wales	South Australia	F Value/U Value	*p*-Value
Constructs				
Motivation (0.84)	4.39 (0.61)	3.94 (0.61)	8.62	0.005
Accessibility (0.85)	4.28 (0.53)	3.88 (0.59)	7.73	0.007
Single items				
There was a good mix of pictorial, text and audio materials in the teaching package	4 (4–4)	4 (3–4)	2.25	0.02
The duration of the lessons was appropriate	4 (4–4)	3 (2–4)	1.68	0.009
